# Human Extraintestinal Sarcocystosis: What We Know, and What We don’t Know

**DOI:** 10.1007/s11908-015-0495-4

**Published:** 2015-06-27

**Authors:** V. C. Harris, M. van Vugt, E. Aronica, G. J. de Bree, C. Stijnis, A. Goorhuis, M. P. Grobusch

**Affiliations:** Center of Tropical Medicine and Travel Medicine, Department of Infectious Diseases, Division of Internal Medicine, Academic Medical Center, University of Amsterdam, Amsterdam, The Netherlands; Amsterdam Institute for Global Health and Development, Amsterdam, The Netherlands; Department of (Neuro)Pathology, Academic Medical Center and Swammerdam Institute for Life Sciences, Center for Neuroscience, University of Amsterdam, Amsterdam, The Netherlands

**Keywords:** Sarcocystosis, *Sarcocystis nesbitti*, Malaysia, Myositis, Sentinel surveillance

## Abstract

There are over 150 known *Sarcocystis* species, and at least one is capable of infecting and causing disease in man. Extraintestinal (muscular) sarcocystosis and intestinal sarcocystosis are the two known manifestations of disease in humans. In this series of six cases and review, we focus on the invasive extraintestinal (“muscular”) form of sarcocystosis in humans. This disease, which until recently was rarely described, has become relevant particularly as an imported condition in travelers due to a recent series of outbreaks reported from Malaysia. Human intestinal sarcocystosis is ubiquitous across the globe. However, absolute numbers of probable and particularly confirmed cases are few, with only several hundred described to date. Characteristically, patients exhibit signs and symptoms either 1–2 weeks after exposure, or after 4–8 weeks. Whether people remain asymptomatic or develop disease apparently depends on the infecting species, host factors, and the inoculum size. The definitive host(s) remain uncertain, and identification of the animal reservoir(s) requires further research. A better understanding of the epidemiology of the disease, as well as its immunological determinants, is hampered by the lack of reliable serological diagnostic methods. Additionally, DNA seems to be contained very effectively within the encysted parasite, thereby rendering PCR detection unreliable. Physicians should suspect the condition in patients with suggestive symptoms and a possible history of exposure. Surveillance networks for imported infectious diseases are formidable tools to help detect and localize outbreaks.

## Biology, Epidemiological, Immunological, and Clinical Features of Human Extraintestinal Sarcocystosis in Brief

Out of the more than 150 known *Sarcocystis* species identified so far, only one, *Sarcocystis nesbitti*, has been proven to date of being capable of infecting and causing disease in man, though others may exist. Human extraintestinal (muscular) sarcocystosis is one of the two forms of illness man may develop following ingestion of, and subsequent infection with one of the species pathogenic to man [1••].

*Sarcocystis* spp. belong to the phylum of the *Apicomplexa* (shared, e.g., with *Toxoplasma gondii* and *Plasmodium* spp.) and are widely distributed throughout the world [1••]. This organism requires a two-host predator (definitive host)-prey (intermediate host) interaction to complete its life cycle. The range of definitive hosts (those in which sexual replication takes place in the gastrointestinal tract, resulting in intestinal sarcocystosis) is broad and includes a variety of carnivorous vertebrates, including humans. Following consumption of sarcocyst-containing flesh, motile bradyzoites released in the small intestine by digestion enter the villous epithelium. Within enterocytes, sexual replication occurs, leading to the formation of sporocysts, which are passed in the feces. Sporocysts ingested by a susceptible intermediate host from fecally contaminated food or water (typically herbi/omnivorous mammals) undergo asexual phases of regeneration. Most classes of vertebrates (including mammals, birds, reptiles/amphibians, and possibly fish) [1••] can be suitable intermediate hosts. Humans can also be infected in this manner, serving as aberrant intermediate hosts. In the intermediate host, the sporocysts excyst in the small intestine. Schizonts develop within blood vessels, and the evolving merozoites invade skeletal, cardiac, and smooth muscles and develop into sarcocysts; in case of infection of humans, we speak of extraintestinal or muscular sarcocystosis. However, sarcocysts can also be found sporadically in neural tissues of some animals [1••].

Human intestinal sarcocystosis has been reported from all regions of the world except Africa and the Middle East. Absence from these regions is most likely related to underdetection and underreporting rather than absence of *Sarcocystis* spp. potentially pathogenic to man. Signs and symptoms of intestinal sarcocystosis are usually limited to acute and rarely chronic gastrointestinal symptoms. Whether people remain asymptomatic or develop some degree of disease eventually requiring treatment (sulfa compounds and others have been tried) may depend on the infecting species, host factors, and inoculum size. Diagnosis of intestinal sarcocystosis is based on coprological examination techniques aimed at direct pathogen detection from feces; molecular detection methods have played a limited role so far [2••]. Two excellent recent comprehensive reviews describe all relevant aspects of intestinal sarcocystosis [1••, 2••].

Below, we will be focusing on the invasive extraintestinal (“muscular”) form of the disease in humans, as interest in this till recently rarely encountered condition has grown as a result of a recent series of outbreaks reported from islands in Malaysia (among others [3••, 4••, 5]). Given the extraordinary number of known *Sarcocystis* species in conjunction with a formidably wide range of definitive and intermediate hosts, it is not surprising that (similar to intestinal sarcocystosis) muscular sarcocystosis cases have been reported from around the world. However, for uncertain reasons, most cases are from Malaysia, with fewer cases from Africa and none from the Middle East. In the case of extraintestinal human disease, in addition to lack of detection as a possible explanation, prevalence of *Sarcocystis* species capable of infecting humans as the intermediate host in specific regions could be important. With clearly less than 100 cases having been reported prior to the recent outbreaks (an excellent synopsis of early cases had been compiled by Beaver and colleagues [6•], while all more recent individual cases and outbreaks have been summarized by Fayer et al. [1••]), the dimension of the problem appeared to be clearly limited in size, if not geographically. Preceding the recent outbreaks, there was evidence that the Malaysian archipelago is apparently a focus of transmission to, and hence disease activity among humans, mostly if not exclusively involving *S. nesbitti*.

Clinical signs and symptoms of symptomatic muscular sarcocystosis are not well characterized. Of the 100 cases of muscular sarcocystosis reported in the literature before the recent outbreaks in Malaysia, no more than 10 symptomatic individuals had been described. An additional 157 symptomatic persons have since been identified as part of the outbreaks among tourists to islands in Malaysia. It is important to recognize that most symptomatic cases reported so far have been among expatriates and travelers, suggesting a role of host immunity in modulating disease manifestations in high-prevalence regions. Disease manifestations may also depend on the infecting species and the infective load. As summarized by Fayer and colleagues [1••], clinical suspicion of muscular sarcocystosis should be raised when encountering a patient with travel or residency history in Southeast Asia, particularly in Malaysia, in combination with fever and/or myalgia, arthralgia, headache, fatigue, and a combination of less frequent symptoms such as a rash. These represent compatible symptoms that might be encountered in an early phase of disease. Later-phase disease may include more severe acute (often circumscribed) myositis typically located in the thighs or the facial/cervical region, often hallmarked by pain and swelling. However, this myositis can occur in virtually all skeletal muscles and (relative to the limited number of cases) also fairly commonly in cardiac muscle.

As it will be described, diagnosis remains challenging, and only the direct demonstration of sarcocysts in muscle offers proof of infection. Molecular methods via PCR have limited sensitivity and serological methods have been unreliable to date [2••].

Not unexpected, with an extremely limited number of patients, no proper therapeutic trials could be conducted. Treatment has included the administration of albendazole for 7–14 days, with or without concurrent steroid [1••], but reported responses may merely have reflected the natural course of the disease. Treatment with trimethoprim/sulfamethoxazole (with known antiprotozoal activity) early in the course of illness may be promising as a potential therapy. However, in the absence of good diagnostic criteria or laboratory testing and adequate therapeutic trials, utility remains unproven. Adjuvant treatment with corticosteroids and nonsteroidal anti-inflammatory medications may help ameliorate symptoms.

Little is known about the immunology and the particularities of the host-parasite interplay of *Sarcocystis* spp. causing extraintestinal disease in man. This is understandable, given the limited number of cases described so far. What is evident from reported cases is that human infection in susceptible individuals elicits a strong eosinophilic and T cell-mediated response. IgG production appears to be unreliable, or difficult to detect reliably; in any case, no convincing studies have been conducted, and seroconversion in cases studied appears to be erratic and hampered by cross-reactivity. The paucity of clinical disease among residents in areas where outbreaks have been noted amongst foreigners suggests that the infection stimulates a humoral (yet T-cell-mediated) memory. However, there is only one single case report in the literature [7] on an AIDS patient in an advanced stage of immunosuppression with concomitant sarcocystosis, manifesting itself atypically as an acalculous cholecystitis; suggesting, apart from an eosinophilic reaction, a role for T-cell-mediated defense. In immune-competent individuals developing disease, the average cytokine production seems to decrease as a sign of early-phase immunosuppression. In the later stage associated with myositis, pro-inflammatory and chemotactic cytokines have been found to be elevated in a series of travelers returning ill from Tioman Island [8].

To fully understand the characteristic epidemiology of the disease, with apparently few cases in local communities in epidemiological foci but rather impressive attack rates in expatriates/travelers to prevalence hotspots, further studies are needed.

## An Overview of the Recent Pangkor and Tioman Island Outbreaks

Two recent outbreaks of symptomatic extraintestinal sarcocystosis rapidly increased the number of documented cases and served to deepen our knowledge of the clinical spectrum of extraintestinal disease, but also highlighted our continuing knowledge gaps. Unanswered questions continue to be the exact risks and modes of transmission, the immunology of the disease as determined in the host/pathogen interplay, and the optimal methods of diagnosis and treatment.

Italiano and colleagues [4••] reported on a group of 92 college students and their teachers who had been in a retreat on Pangkor Island, off the west coast of the Malaysian peninsula, in January 2012. Of the 92, 89 (97 %) fell ill within 26 days, the majority within 9–11 days. The most likely common source of exposure was contaminated drinking water. Most cases had fever (94 %) and myositis (91 %) as well as headaches (87 %), with less than 50 % of patients complaining of arthralgia or gastrointestinal symptoms. Of note, nine patients (10 %) suffered from transient swelling of masseter muscles; only a few exhibited a transient rash (5 %). The median duration of symptoms was 17 days, with non-Malaysian patients requiring a longer time period to recovery than Malaysians. In an early stage of disease, 32 % of patients had elevated eosinophil counts, 60–70 % yielded moderately elevated liver enzymes, and a minority (15 %) had an elevated creatine phosphokinase (CPK). In a later phase, i.e., more than 4 weeks after the first possible time point of exposure, eosinophilia was present in 67 % of cases, and 90 % of those tested had an elevated CPK, while the proportion of patients with moderately elevated liver enzymes remained more or less unchanged. Sarcocysts were identified in three out of four muscle biopsies, with PCR speciation yielding *S. nesbitti* in all three cases. On the grounds of shared clinical features and exposure, it was postulated that all patients likely had extraintestinal sarcocystosis, rendering this the biggest outbreak of extraintestinal sarcocystosis reported to date. Symptoms resolved spontaneously in the vast majority of patients. Two patients received corticosteroids, and in one patient, a specific treatment attempt with sulfadoxine/pyrimethamine plus clindamycin was made, without a clear correlation between treatment and improvement of symptoms or normalization of laboratory values.

Serological diagnosis was attempted on paired sera from ten patients including those who were classified as “definite sarcocystis” due to positive microscopy and/or PCR results; in one of those cases, serology was negative.

The exposure investigation suggested accidental use of contaminated untreated water from a nearby stream for preparation of food and drink at the hotel where the retreat took place.

Beginning early in 2011, and within 2 years, an increasing number of travelers returning to their overseas home countries presented with signs and symptoms of what soon was suspected to constitute an outbreak of muscular sarcocystosis amongst travelers to Tioman Island, off the east coast of the Malaysian peninsula [3••, 5, 9–11]. Applying the case definition given in Table 2, 62 probable case patients and six patients confirmed by histological proof or DNA isolation from muscle biopsy were seen in Europe, Canada, and Singapore with complaints of myalgia (100 %), fatigue (91 %), fever (82 %), and less frequently headache (59 %) and arthralgia (29 %), with a distinct clustering into early (2 weeks after departure from the island) and late (6 weeks after) phases, and with CPK and blood eosinophilia levels rising distinctively in the second phase during the fifth week after departure.

The definitive diagnosis depends upon muscle biopsy, and not even regularly so, as in only 6 out of 15 muscle biopsies, sarcocysts were found. Ten patients underwent PCR testing of their muscle biopsies, of which one (also positive on histology) was positive and identified as *S. nesbitti* by sequence analysis. Only a minority of patients could be definitely confirmed, rendering all other patients probable cases. No alternative diagnoses could be established. Although identification of a common source of infection was not possible, all but one patient was aware of potential exposure to untreated water. Tappe and colleagues [12•] reviewed sarcocystosis in animals in Malaysia and provide a comprehensive list of potential and confirmed intermediate wild and domestic host species. A resident snake species (the Indian or spectacled cobra (*Naja naja*) has been suggested to be a natural definitive host for *S. nesbitti* [13–15]. Others such as the reticulated python have been identified as definitive host for animal-infecting *Sarcocystis* spp. [12•]), and for the time being, water contamination appears to be the most likely source of infection. Of note, an environmental investigation of the Malaysian Ministry of Health did not recover any *Sarcocystis* species from sampled water sources in the most afflicted region [16].

## A Closer Look at a Series of Extraintestinal Sarcocystis Patients Encountered in the AMC Tropencentrum

Between 2011and 2014, six patients presented to our outpatient traveler’s clinic after a trip to Malaysia. They all had visited the island of Tioman, Malaysia. All patients were relatively young with no significant medical history. Shared potential risk factors were as follows: contact with sea water and beaches, potential contact with contaminated drink and/or food sources, and recollection of noting undomesticated cats.

After their return, back to the Netherlands, two of the patients developed complaints shortly (within 2 weeks) after exposure and the four others up to 3 months after exposure. The patients developed varying complaints, ranging from fever to general malaise and slowly progressive exhaustion with muscle aches, continued itching without a skin rash, shortness of breath, diminished physical condition, and weight loss.

Physical examination revealed few diagnostic clues; however, most patients appeared fatigued and demonstrated exhaustion during minimal exertion.

Initial laboratory results showed elevated CPK, alanine aminotransferase (ALAT), and lactate dehydrogenase (LDH) and slightly elevated C-reactive protein (CRP) and erythrocyte sedimentation rate (ESR) in combination with an eosinophilia. In two patients, the eosinophilia was not present initially; however, it developed within 2 weeks. Of note, these two patients had highly elevated CPK levels on presentation. In all six patients, further microbiologic testing has been performed and all additional tests performed in selected patients (i.e., not all tests were conducted in all patients—trichinellosis, toxoplasmosis, toxocariasis, strongyloidiasis, schistosomiasis, filariasis, paragonimiasis and cysticercosis, dengue and chikungunya, and rickettsiosis) turned out to be negative. All patients had negative trichinellosis serology. Some imaging studies were performed, all with no abnormalities. Key features of all six patients are summarized in Table 1.

**Table 1:** AMC Tropencentrum patients who visited Tioman Island and returned with symptoms suggestive of acute muscular sarcocystosis

Patient no.	Age	Sex	Underlying medical condition	Pretravel advice	Initial symptoms	Time between onset of symptoms and most likely time point of exposition	Vital signs on presentation	Physical examination findings	Serology/PCR positive sarcocystis	WBC/eosinophils (both 10^9^/L)	CPK (U/L)	Muscle biopsy	Recovery after treatment with albendazole (A)
1	46	M	IBS	No	Malaise, fever, fatigue, myalgia, shortness of breath	3 months	Abnormal: respiratory rate 38/min	Moderately ill	Seronegative	9.2/1.38 (15 % eos)	275	Perivascular inflammatory infiltrate; no sarcocysts seen	1 week A, full recovery
2	44	F	N/A	Yes	Myalgia, skin rash,headache	2 months	Normal	Mildly ill	Seronegative	7.4/0.97^a^	121	Not performed	2 weeks A, full recovery
3	51	F	N/A	Yes	Myalgia, joint pain, fatigue	1.5 months	Normal	Moderately ill	Seronegative	19.4/0.12^a^	176	Not performed	10 days A, full recovery
4	30	F	N/A	Yes	Malaise, fever, fatigue, myalgia	2 weeks	Normal	Mildly ill	PCR negative	6.2/ 0.23 (1 % eos)	677	Perivascular inflammatory infiltrate; cyst seen with bradyzoites	1 week A+ P (+ prednisone), full recovery
5	34	M	N/A	Yes	Malaise, fever, fatigue, myalgia, night sweats, arhralgia	2 weeks	Normal	Moderately ill	PCR negative	5.6/0.18 (0.86 % eos)	843	Perivascular inflamm. infiltrate; no sarcocysts	1 week A + P, full recovery
6	30	F	N/A	Yes	Headache Fever, fatigue, myalgia	1 week 4 weeks	Normal	Mildly ill	Seronegative	5.7/0.51^a^ (mild eosinophilia)	104	Not performed	1 week A, full recovery

IBS irritable bowel syndrome, *N/A* not applicable

^a^Percetnage not determined

**Fig. 1:**
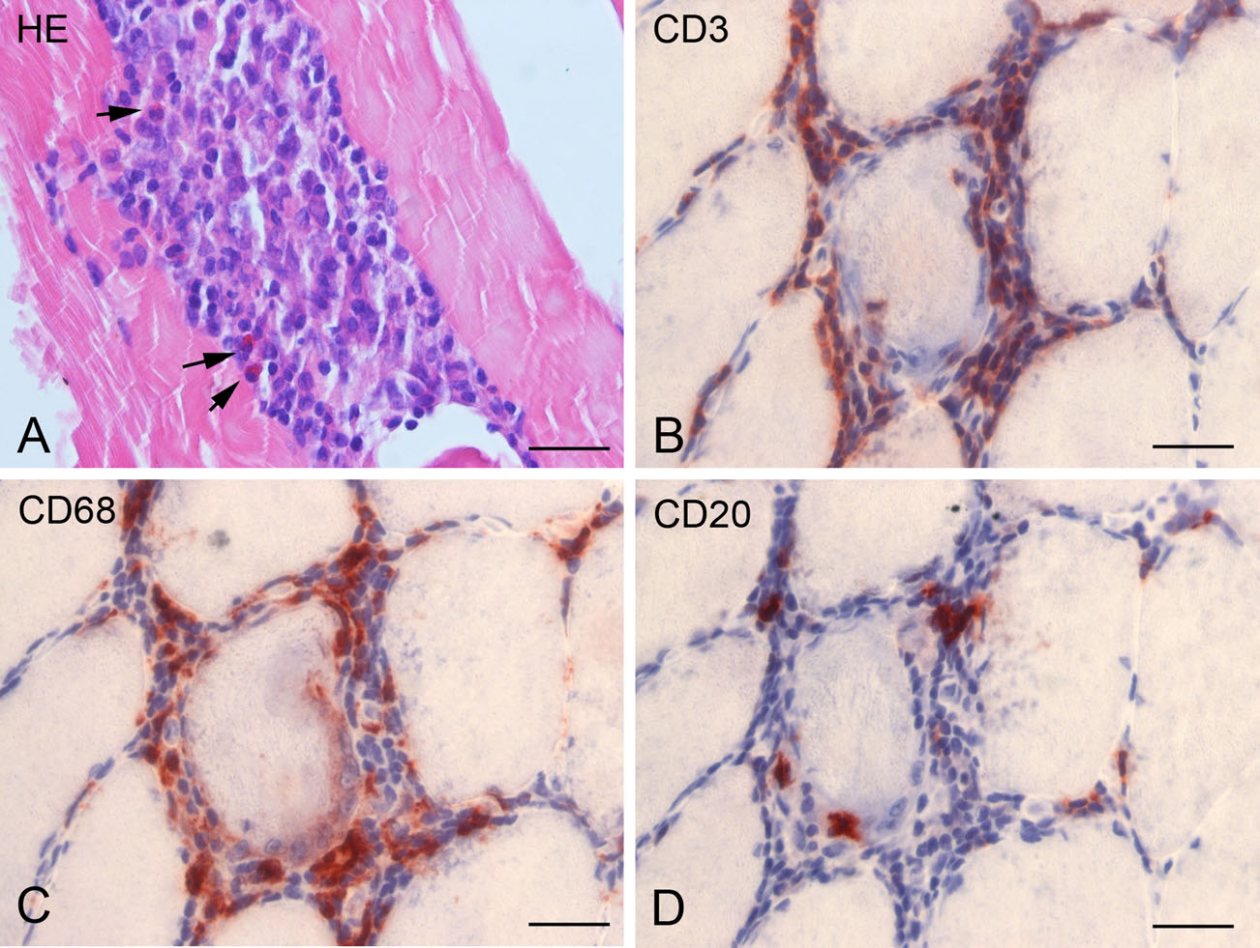
Muscle biopsy of patient #1. **a** Hematoxylin and eosin (HE) stain showing an endomysial inflammatory cell infiltrate with eosinophils (*arrows*). **b** (CD3) T lymphocytes with invasion of muscle fibers. **c** (CD68) macrophages. **d** (CD20) B lymphocytes. *Scale bars*, 40 μM

During our first patient’s evaluation (see Case Vignette), GeoSentinel/EuroTravNet (the Global [17] and European [18] Surveillance Networks of the International Society for Travel Medicine (ISTM)), TropNet (another former surveillance, now research network focusing on Europe) [19] and the Centers for Disease Control (CDC) issued preliminary reports of *Sarcocystis* infection in travelers returning from Tioman Island, Malaysia, after travel between June and August of 2011. Most travelers had complaints of muscle pain, elevated CPK and eosinophilia. Attempting to determine if our patient was suffering from a *Sarcocystis* infection, we performed a muscle biopsy in October 2011, showing myositis with eosinophil infiltration, but not sarcocysts (Fig. 1a–d).

**Fig. 2:**
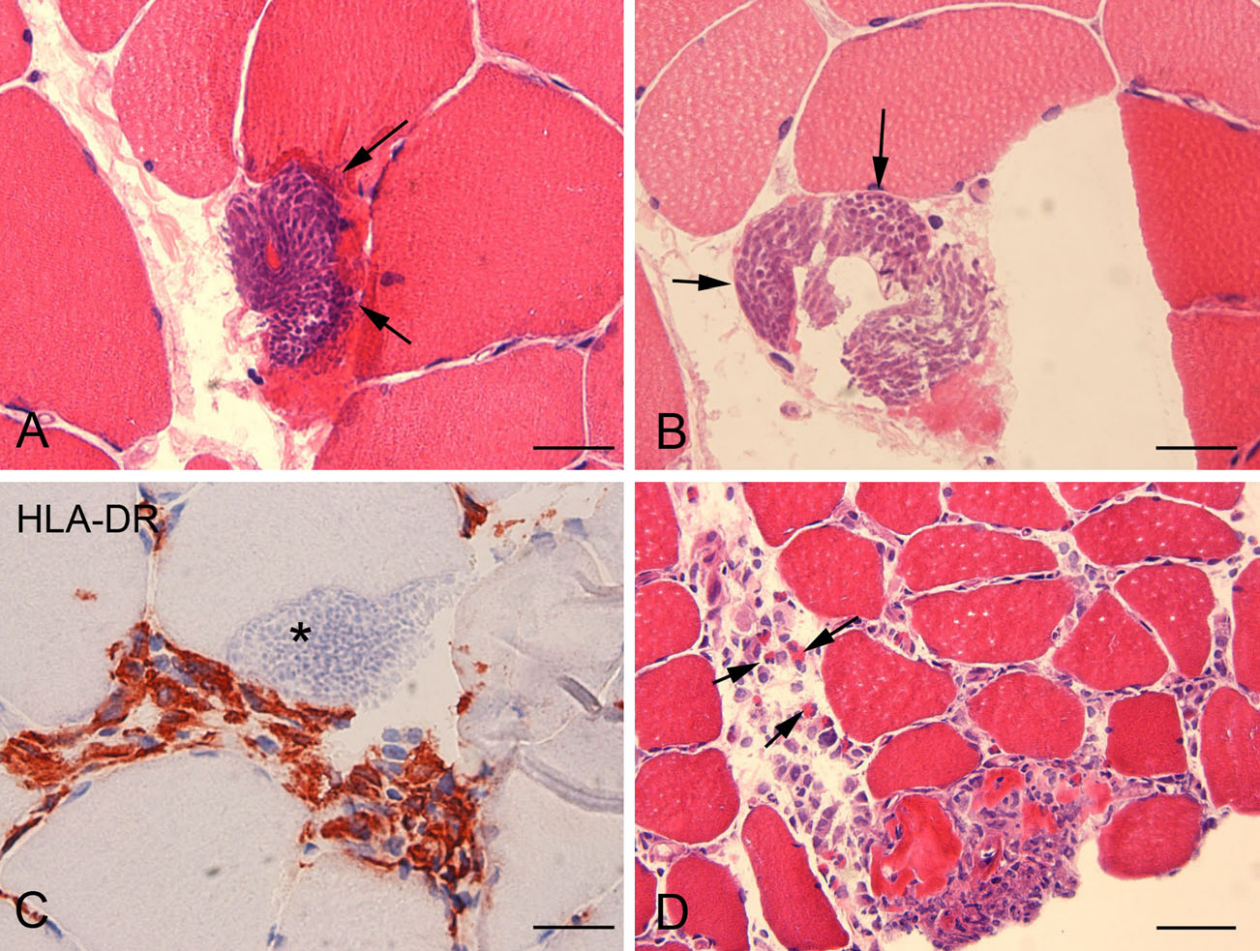
Muscle biopsies of patients #1 and #3. **a**–**b** Hematoxylin and eosin (HE) stain showing sarcocysts within the muscle fibers. **c** Major histocompatibility complex class II (MHC II): inflammatory cell infiltrate around sarcocyst (*asterisk*). **d** HE stain showing endomysial inflammatory cell infiltrate with eosinophils (*arrows*). *Scale bars*, **a**–**c** 40 μM; **d** 160 μM

Muscle biopsies were also performed on two other patients. In one patient, the biopsy showed the presence of sarcocysts without prominent infiltrates, whereas in the other patient the biopsy showed myositis with eosinophil infiltration. but not sarcocysts in situ (Fig. 2a–d).

According to the Geosentinel and CDC case definition for *Sarcocystis* (see Table 2), our patients had probable acute muscular sarcocystosis. All were empirically treated with albendazole 400 mg BID/7–10 days (two of them received prednisone, 20 mg orally over 5 days each), and all recovered with complete resolution of all symptoms and normalization of the laboratory values, with no alternative diagnoses determined.

**Table 2:** Definition of probable and confirmed sarcocystosis as handled by GeoSentinel and CDC

Probable acute muscular sarcocystosis
- Travel to Tioman Island after March 1, 2011
- Myositis (requiring at least one of the following):
▪ A complaint of muscle pain and a CPK level >200 international units per liter (IU/L)
▪ Muscle tenderness documented on physical examination
▪ Histologic evidence of myositis in a muscle biopsy
- Eosinophilia >500 cells per microliter (cells/μL)
- Negative *Trichinella* serology
Confirmed acute muscular sarcocystosis
- Histologic observation of intramuscular cysts compatible with sarcocysts or the isolation of *Sarcocystis* spp. DNA from a muscle biopsy.

Source: Esposito et al. [3••]

### Case Vignette

Mr. B was a 48-year-old man who presented to us approximately 3 months after a trip to Malaysia with complaints of muscle pain, itching, and shortness of breath.

The patient’s past medical history was significant only for an appendectomy and the diagnosis of irritable bowel syndrome 3 years prior to presentation. He had been vaccinated against diphtheria, tetanus and pertussis, hepatitis A, and *Salmonella typhi* several months prior to his travel.

The patient began his travels on 19 July 2011 when he flew from Amsterdam, the Netherlands, to Dubai in the Arab Emirates with his two children. They spent 2 days enjoying the beach and shopping before continuing on to Bangkok, Thailand, on 21 July. In Bangkok, the patient visited temples and ate local food. On 27 July, the family flew through Kuala Lumpur and onto the island of Tioman, Malaysia. There they stayed in a hotel resort for 9 days. They spent the majority of their time on the beach and swam and snorkeled in salt water. The patient flew back to Amsterdam on August 4, 2011.

On August 20, 2011—2 weeks after his return to Amsterdam—he developed general malaise and slowly progressive exhaustion. Around September 10—a little more than 1 month after his return—he noted night sweats, generalized pruritus, and muscle aches throughout his entire body. On September 20—6 weeks after his return—he developed a fever up to 38.5 °C and noticed shortness of breath on exertion and dizziness upon standing. On October 4—2 months after his return—he presented to our travelers outpatient clinic with continued pruritus without a skin rash, muscle aches, shortness of breath on minimal exertion, postural dizziness, occasional stabbing chest pain, and 2 kg of weight loss.

On physical examination, the patient was moderately ill-appearing with tachypnea to 28 breaths per minute on exertion. His oxygen saturation was 100 % on room air, sitting blood pressure was 110/70 mmHg with a pulse of 60 beats per minute and dropped to 90/64 mmHg on standing with a pulse increase to 96 bpm. The remaining physical examination was completely normal except for pain on deep palpation of the right upper quadrant without rebound tenderness.

Initial laboratory results were as follows: CRP 25 mg/L; bilirubin 5 μmol/L; aspartate aminotransferase (ASAT) 33 U/L; ALAT 55 U/L; alkaline phosphatase 60 U/L; LDH 271 U/L; CPK 275 U/L; leukocytes 9.2 × 10^9^/L (eosinophils 1.38 × 10^9^/L = 15 %); hemoglobin (Hb) 10.0 mmol/L; mean corpuscular volume (MCV) 87 fL; thrombocytes 31 × 10^9^/L; D-dimers 2.50 mg/L). CK-MB (muscle-brain) and cardiac troponin measurements were negative.

Imaging studies were performed. A chest X-ray and CT of the thorax were performed—these showed no pulmonary infiltrates, no sign of a pulmonary embolism, and no lymphadenopathy. An abdominal echo demonstrated a normal liver, gallbladder, spleen, and kidneys. An ECG showed sinus rhythm, normal conduction, and no signs of hypertrophy or ischemia. Because of the patient’s continued complaints of exertional dyspnea, a cardiac echo was performed which showed normal left ventricular systolic function, a dilated right ventricle with a minimal tricuspid insufficiency.

Microbiologic test included triple feces tests (twice); blood cultures (twice), and *Strongyloides*, *Fasciola*, *Filaria*, *Toxocara*, *Trichinella*, *Toxoplasma*, HIV, CMV, and EBV serologies. Except for a positive IgG for EBV, all tests/serologies were negative. Since more than 6 weeks had passed after return, the patient presented beyond the time frame to consider dengue or chikungunya virus infection.

During the patient’s evaluation, the GeoSentinel (the Global Surveillance Network of the International Society for Travel Medicine (ISTM) program director gave a preliminary report of *Sarcocystis* infection in travelers returning from Tioman Island, Malaysia, after travel between June and August of 2011. Most travelers had complaints of muscle pain, elevated CPK and eosinophilia, with many features shared with our patient. In order to determine whether our patient was suffering from a *Sarcocystis* infection, we performed a muscle biopsy in October 2011.

The muscle biopsy (Fig. 1) showed inflammatory cell infiltrate within the endomysium composed of eosinophils, T lymphocytes (with invasion of muscle fibers), macrophages, and few B lymphocytes. The muscle biopsy tissue blocks were subsequently shipped to the CDC Infectious Diseases Pathology Branch for additional examination, including histopathology, polymerase chain reaction (PCR) detection, and DNA sequencing analysis of *Sarcocystis* spp. 18S rRNA amplicons. These were all negative. However, given the highly unusual finding of an eosinophilic myositis (also found in the other two cases in whom biopsy was performed), the pathology reports were interpreted as highly suggestive for an invasive sarcocystis infection.

Our patient was empirically treated with 7 days of albendazole 10 weeks after return, and by 14–16 weeks after his return to the Netherlands, he had complete resolution of all symptoms and normalization of both his eosinophilia and his CK. It is uncertain if his recovery was representative of the normal course of disease or perhaps influenced by the albendazole. A last interesting detail in the course of his disease was the development of a subcutaneous nodule on his left calf 20 weeks after his return, with spontaneous resolution, and as described before in one of the patients from the Tioman Island case series [3••].

According to the recent GeoSentinel and CDC case definition for sarcocystis [3••] (Table 2), our patient had probable acute muscular sarcocystosis. In the recent Tioman Island case series review [3••], patient symptoms seemed to occur in two distinct phases—early (after the second week of exposure) and late (after the sixth week of exposure). This patient’s symptoms began 2 weeks after his return from Tioman and he appeared to have peak muscle complaints approximately 6 weeks after his return, suggesting a possible immune-mediated myositis. His case is particularly interesting because of his predominant complaints of dyspnea on exertion and chest pain in combination with postural hypotension and a dilated right ventricle on echocardiography, suggesting possible *Sarcocystis* or immune involvement of his heart; and the subcutaneous nodule appearing and resolving spontaneously.

This overall small series of patients of which patient #1 was included in the Esposito et al. case compilation [3••] highlights many of the common features of probable muscular sarcocystosis but also highlights the diagnostic difficulties encountered in confirming the diagnosis.

## Human Sarcocystosis: What Are the Knowledge Gaps?

Although the latest outbreaks contribute significantly to our knowledge, several matters remain unresolved. Firstly, although it is apparent that *S. nesbitti* seems to form a prevalence epicenter around the Malaysian peninsula, the global epidemiology of (particularly) extraintestinal sarcocystosis remains patchy and unclear. With a “supershedder” remaining unidentified but suspected in Indian cobras and maybe other snakes, a single definitive host may be responsible for high numbers of patients in very circumscribed areas. It would be of great interest with possible public health implications to pursue identification of this (or multiple, regionally highly infested) animal reservoir(s). With *Sarcocystis* species definitely or potentially infectious to man as an aberrant intermediate host, it remains unclear whether sporadic cases or even epidemic clusters were missed due to plausible alternative diagnoses (or ignorance), or whether geographical distribution is as distinct as it appears.

Of note, the fascinating immunology and host/parasite interplay remain by-and-large to be elucidated, which is much needed to fully understand the epidemiologic characteristics of the disease, with few cases in local populations but outbreaks among expatriates/travelers to particular foci. Better understanding of the epidemiology of the disease is of utmost importance as there is evidence that the local population [2••] is probably more frequently able to contain infection at subclinical levels. However, this hampered by the lack of reliable diagnostic methods. To that end, *Sarcocystis* species pose formidable diagnostic challenges in extraintestinal disease. At this point in time, there is little evidence that this will be easily overcome in the near future, pinpointing the need for a high level of clinical suspicion, and exclusion of potential differential diagnoses, to reliably identify patients. It has been shown that in situations outside a clustering in a “real” outbreak such as on Pangkor Island, surveillance networks for imported infectious diseases are powerful tools to detect hyperendemic areas or ongoing outbreaks. Surveillance networks are also of particular importance in detecting cases of this disease that appears to be more pronounced in travelers than in locals. It appears to be wishful for the sake of the patients that physicians include, despite the paucity of cases, extraintestinal sarcocystosis in their differential diagnosis of patients with a possible history of exposure, a suggestive clinical picture of myositis and accompanying signs and symptoms.

## References

[1.••] Fayer R, Esposito DH, Dubey JP (2015). Human infections with *Sarcocystis* species. Clin Microbiol Rev.

[2.••] Poulsen CS, Stensvold CR (2014). Current status of epidemiology and diagnosis of human sarcocystosis. J Clin Microbiol..

[3.••] Esposito DH, Stich A, Epelboin L, Malvy D, Han PV (2014). Acute muscular sarcocystosis: an international investigation among ill returned travelers returning from Tioman Island, Malaysia, 2011-2012. Clin Infect Dis..

[4.••] Italiano CM, Wong KT, AbuBakar S, Lau YL, Ramli N (2014). Sarcocystis nesbitti causes acute, relapsing febrile myositis with a high attack rate: Description of a large outbreak of muscular sarcocystosis in Pangkor Island, Malaysia, 2012. PLOS Neglected Tropical Diseases.

[5] Tappe D, Stich A, Langeheinecke A, von Sonnenburg F, Muntau B, et al. Suspected new wave of muscular sarcocystosis in travellers returning from Tioman Island, Malaysia, May 2014. Eurosurveill. 2014;19(21) 10.2807/1560-7917.es2014.19.21.20816 24906376

[6.•] Beaver PC, Gadgil RK, Morera P (1979). *Sarcocystis* in man: a review and report of five cases. Am J Trop Med Hyg..

[7] Agholi M, Heidarian HR, Moghadami M, Hatam GR (2014). First detection of acalculous cholecystitis associated with Sarcocystis infection in a patient with AIDS. Acta Parasitol..

[8] Tappe D, Slesak G, Pérez-Girón JV, Schäfer J, Langeheinecke A, Just-Nübling G et al. Human invasive muscular sarcocystosis induces Th2 cytokine polarization and biphasic cytokine changes – an investigation among returning travelers from Tioman Island, Malaysia. Clin Vacc Immunol. 2015 [*Epub ahead of print]* 10.1128/CVI.00042-15 PMC4446401 25903356

[9] Centers for Disease Control and Prevention (2012). Notes from the field: acute muscular sarcocystosis among returning travelers – Tioman Island, Malaysia, 2011. MMWR Morb Mortal Wkly Rep..

[10] Esposito DH, Freedman DO, Neumayr A, Parola P. Ongoing outbreak of an acute muscular *Sarcocystis*-like illness among travellers returning from Tioman Island, Malaysia, 2011-2012. Euro Surveill. 2012;17. PMC4620707 23153473

[11] Tappe D, Ernestus K, Rauthe S, Schoen C, Frosch M, Müller A (2013). Initial patient cluster and first positive biopsy findings in an outbreak of acute muscular Sarcocystis-like infection in travelers returning from Tioman island, Peninsular Malaysia, in 2011. J Clin Microbiol..

[12.•] Tappe D, Abdullah S, Heo C, Kannan Kutty M, Latif B (2013). Human and animal invasive muscular sarcocystis in Malaysia—recent cases, review and hypotheses. Trop Biomed..

[13] Tian M, Chen Y, Wu L, Rosenthal BM, Liu X (2012). Phylogenetic analysis of *Sarcocystis nesbitti* (*Coccidia*: *Sarcocystidae*) suggests a snake as its probable definitive host. Vet Parasitol..

[14] Lau YL, Chang PY, Subramaniam V, Ng YH, Mahmud R, Ahmad AF, Fong MY (2013). Genetic assemblage of *Sarcocystis* spp. in Malaysian snakes. Parasit Vectors..

[15] Lau YL, Chang PY, Tan CT, Fong MY, Mahmud R, Wong KT (2014). *Sarcocystis nesbitti* infection in human skeletal muscle: possible transmission from snakes. Am J Trop Med Hyg..

[16] Husna Maizura AM, Khebir V, Chong CK, Shah A, Hakim L (2012). Surveillance for sarcocystosis in Tioman Island. Malaysia. Malaysian J Public Health Med..

[17] http://www.istm.org/geosentinel [last accessed on 10 May 2015]

[18] http://www.istm.org/eurotravnet. Accessed 10 May 2015

[19] http://www.tropnet.net. Accessed 10 May 2015

